# Identification of a Novel *GRM1* Frameshift Variant in Two Pakistani Families Broadens the Genetic Landscape of Ultra-Rare Spinocerebellar Ataxia Type 13

**DOI:** 10.1007/s12311-025-01897-w

**Published:** 2025-08-27

**Authors:** Riaz Ahmad, Mina Zamani, Eleanor Self, Salah Ud Din Shah, Muhammad Naeem, Henry Houlden

**Affiliations:** 1 Medical Genetics Research Laboratory, Department of Biotechnology, Quaid-I-Azam University, Islamabad, 45320 Pakistan; 2 Department of Neuromuscular Disorders, UCL Queen Square Institute of Neurology, Queen Square House, London, WC1N 3BG UK; 3 Department of Biotechnology and Genetic Engineering, Kohat University of Science and Technology, Kohat, Pakistan

**Keywords:** Neurodegenerative disorders, Intellectual disability, Whole exome sequencing, Glutamate metabotropic receptor 1, Clinical heterogeneity

## Abstract

Autosomal recessive spinocerebellar ataxia 13 (SCAR13) is an extremely rare neurodegenerative disorder characterized by psychomotor delay, ranging from mild to severe intellectual disability with absent or poor speech development, nystagmus and stance ataxia. If ambulation is achieved, affected subjects often exhibit gait ataxia. Additionally, epilepsy and polyneuropathy have been reported in some patients. SCAR13 is caused by pathogenic variants in the *GRM1* gene, which is predominantly expressed in the cerebellum, with lower levels in the other parts of the brain. To date, only seven reports of this rare ataxia have been published globally. Our study aimed to investigate clinical and mutation spectrum of *GRM1*-associated SCAR13 disorder in nine patients of two consanguineous Pakistani families (designated here to as NP35 and NP36). We performed whole exome sequencing in the probands of the two families followed by Sanger sequencing to test variant segregation. We identified a novel *GRM1* frameshift variant (NM_001278064.2):c.3525_3529del; p.(Asn1176IlefsTer71) in both families as a cause of SCAR13. It was classified as a variant of uncertain significance (PM2: pathogenic moderate 2 and PVS1: pathogenic very strong 1) according to the ACMG guidelines. The novel variant exhibited clinical heterogeneity in the two families. Moreover, scoliosis was observed in all four patients of the family NP35, a feature previously documented in only one patient worldwide. Our study expands the limited mutation spectrum of the *GRM1*-associated SCAR13. Next-generation sequencing plays a pivotal role in the elucidation of inherited neurological disorders and in a better understanding of the convergent phenotypes.

**Supplementary Information:**

The online version contains supplementary material available at 10.1007/s12311-025-01897-w.

## Introduction

Autosomal recessive spinocerebellar ataxia 13 (SCAR13; MIM 614831) is an ultra-rare disorder caused by pathogenic or disruptive mutations in the *GRM1* gene, located on chromosome 6q24.3 [31]. *GRM1* encodes the metabotropic glutamate receptor 1 (mGluR1) protein, which is a G-protein coupled receptor and plays a role in neuronal excitability, synaptic plasticity, and feedback inhibition of neurotransmitter release. SCAR13 is a neurological condition characterized by delayed psychomotor and abnormal intellectual development starting in infancy. Other features include poor or absent speech, hyperreflexia, as well as gait and stance ataxia. Most subjects also exhibit abnormalities in eye movements [16]. In 2012, Guergueltcheva et al. first reported the *GRM1* gene associated with SCAR13 by two biallelic variants in five Roma families, and later in 2015, Davarniya et al. reported a second novel biallelic variant in a single Iranian family. All these patients had intellectual disability and cerebellar ataxia.

So far, only seven reports of the *GRM1* gene variants associated with SCAR13 have been published, as summarized in Table 1. SCAR13 (MIM:614,831) associated *GRM1* variants are null variants, leading to the loss of mGluR1 function [28]. These reported variants included three missense, two nonsense, two deletions, and one splice site variant [10, 23, 24]. Pathogenic variants in *GRM1* are also linked to autosomal dominant spinocerebellar ataxia 44 (SCA44; MIM: 617691), which is less severe than SCAR13 phenotype [29].

**Table 1:** A comparison of clinical manifestations of SCAR13 patients with *GRM1* (NM_001278064.2) variants as reported in the literature

Features	[16]	[10]	[7]	24	[31]	[23]	[8]	Present Study (Family NP-35 & NP-36)
No of Patients Reported	10	3	1	3	3	1	1	9
MAF	NA	NA	exomes: ƒ = 0.000000684 genomes: not found	exomes: ƒ = 0.000000684 genomes: ƒ = 0.00000657	NA	NA	NA	NA
cDNA Variant	c.2652_2654del and c.26660 + 2 T > G	c.1360C > T	c.889C > T	c.718G > A	c.718G > T	c.2471C > G	c.1258_1297del and c.2368_2369del	c.3525_3529del p.(Asn1176IlefsTer71)
Zygosity	Homozygous	Homozygous	Homozygous	Homozygous	Homozygous	Homozygous	Compound Heterozygous	Homozygous
Variant Type	Deletion/Splice site	Missense	Nonsense	Missense	Nonsense	Missense	Deletion	Deletion
Onset	Early	Early	Early	Early	Early	Early	Late	Early
Developmental Delay	+	+	+	+	+	+	+	+
Intellectual Disability	+ (Intellectual disability)	+ (Moderate to severe)	+ (Severe/global)	+ (Moderate to severe)	+ (Severe)	-	+	+
Speech Impairment	+	+	+	+	+	NA	+	+
Cerebellar Atrophy/Hypoplasia	+	+	+	-	+	+	-	+
Gait/Stance Ataxia	Gait/stance ataxia	Gait/stance ataxia	No walking	Gait ataxia	Quadrupedal gait	Gait/stance ataxia	No independent walking	No independent walking
Dysarthria	+	-	-	-	+	+	-	+
Seizures	-	+	-	-	-	-	-	Dystonic tremors and seizures (in NP35) and Mild involuntary movement (frequent falls)
Nystagmus	-	+	+	-	-	-	+ (Mild)	Nystagmus in family NP35
Pyramidal Signs	+ (Mild)	-	+	-	+	-	-	+
Scoliosis	-	-	-	-	+	-	-	+ (NP-35)
Behavioral Issues	-	+ (Aggressive)	-	+ (Aggressive)	+ (Aggressive)	-	-	NA
Other Features	Inferior vermian hypoplasia, dysdiadochokinesi,dysmetria	Esotropia (in one patient), short stature	Autism spectrum disorder, axial hypotonia, joint hyperlaxity	Spasticity, eye abnormalities, dental and skeletal anomalies, ear shape anomalies	Peripheral neuropathy, eye ptosis, strabismus, pes planus, non-progressive	Coarse discoordination when performing dynamic hand tests	Balance disturbance, diffuse hypotonia, mild dysphagia	Balance disturbance

*GRM1* is mainly expressed in the cerebellum [18, 19, 23] but is also present in the spinal cord, cerebral cortex, basal ganglia, medulla oblongata, bone marrow, heart and kidney [31]. It is highly expressed in cerebellar Purkinje cells [21], localized at postsynaptic densities and functions by activating phospholipase C, leading to the formation of inositol 1,4,5-triphosphate/diacylglycerol [11, 14, 30]. Through the activation of second messenger systems, *GRM1* plays a crucial role in cognition, cerebellar development and neuroprotection, thereby maintaining synaptic plasticity [3].

According to [6], *GRM1* gene contains eight exons making mGluR1 monomer composed of extracellular region, including venus flytrap domain (VFTD) and cysteine-rich domain (CRD). Two other domains are 7 transmembrane domains (TM1-TM7) and C-terminal region (CTD). C-terminal domain varies in alpha and beta isoforms of mGluR1 [23]. VFTD [7, 10, 16, 24, 31] and TM7 domains [16] are the most frequently mutated regions associated with SCAR13. So far, mutation in C-terminal domain is not reported in the literature for SCAR13 while documented by Watson et al. [29] for SCA44 disorder.

In the present study, we investigated nine patients from two unrelated Pakistani families affected with diverse manifestations of SCAR13. This study broadens and strengthens our understanding of the genetic defects in the *GRM1* gene, which underlie a variety of clinical symptoms. According to our recent systematic analysis [1], 90% of the reported cases of neurological disorders in the Pakistani population are attributed to consanguineous marriages, as diagnosed through whole exome sequencing (WES). Both families included in this report originate from the same ethnic and geographic region, where the consanguinity rate is so high. Therefore, it may increase the likelihood of rare founder mutations being shared across ostensibly unrelated families.

## Material and Methods

### Human Subjects and Ethical Approval

Our study was approved by the Institutional Review Board of Quaid-I-Azam University, Islamabad. All experiments were undertaken with the understanding and written consent of each subject. The study conforms with the World Medical Association Declaration of Helsinki. Human families (labelled as NP35 and NP36) included in this research study belong to the Khyber Pakhtunkhwa province of Pakistan. Saliva and peripheral blood samples were collected for genomic DNA isolation by using Oragene saliva collection kit (Cat# OG500, DNA Genotek Inc., Ottawa, ON, Canada) and Qiagen DNA extraction kit (Cat# 56,304, QIAamp, Qiagen, Valencia, CA, USA), respectively. Extracted DNA was further quantified through Quantus Fluorometer (Cat# E6150, Promega, Madison, WI, USA).

### Genetic Screening

#### Next-Generation Sequencing

To identify the disease-causing variants, whole exome sequencing was carried out at Macrogen Inc., Korea, through the Agilent SureSelect Human All Exome V6 Kit (Agilent Technologies, Santa Clara, CA, USA) as described previously [12]. The paired-end sequencing (PE150) was performed by the Illumina NovaSeq 6000 (Illumina, Santa Clara, CA, USA). The obtained sequencing reads were aligned against the human reference genome (hg19 reference genome assembly) through BWA (Burrows-Wheeler Aligner v0.7.17: http://bio-bwa.sourceforge.net/bwa.shtml). For the recruited families, the hg19 reference genome assembly was selected, while the hg38 assembly was used against sequencing reads. The BAM files were sorted and the duplicate reads were marked through Samtools (v1.8) (https://github.com/samtools/samtools/releases/tag/1.8) and Picard (v2.18.9) (http://sourceforge.net/projects/picard/), respectively. For genotyping, Genome Analysis Toolkit (GATK: https://gatk.broadinstitute.org) v4.0 was used. For functional annotation, Annotate Variation (ANNOVAR: https://annovar.openbioinformatics.org/en/latest/) was performed, while for variant filtration, FILTUS (http://folk.uio.no/magnusv/filtus.html) was used as described earlier [2]. After proper annotation, the generated file was recovered in the CSV format that was further filtered to identify the possible pathogenic variants.

Our strategy for bioinformatic filtration consisted of screening for exonic sequences and splice acceptor/donor splice sites (Figure S1). According to the phenotypes and pedigrees, we prioritized the rare variants having minor allelic frequency < 0.01% in public databases such as The Genome Aggregation Database (https://gnomad.broadinstitute.org/), 1000 Genomes Project (https://www.internationalgenome.org/), NHLBI Exome Variant Server (http://evs.gs.washington.edu/EVS/) and Complete Genomics 69 (https://www.completegenomics.com/). Secondly, the pattern of inheritance (such as homozygous, and compound heterozygous) and phenotypes (including neuropathy, disability and other neurological disorders) were considered during filtration. Thirdly, the pathogenic impact was considered especially for nonsense, missense, splice site and frameshift variants. Different in silico tools were used for deleterious effects, including CADD score greater than 17, SIFT and Polyphen 2. Furthermore, pathogenicity of the variants was assessed based on the American College of Medical Genetics and Genomics (ACMG) guidelines.

#### Sanger Sequencing and Runs of Homozygosity (ROHs)

**Fig. 1:**
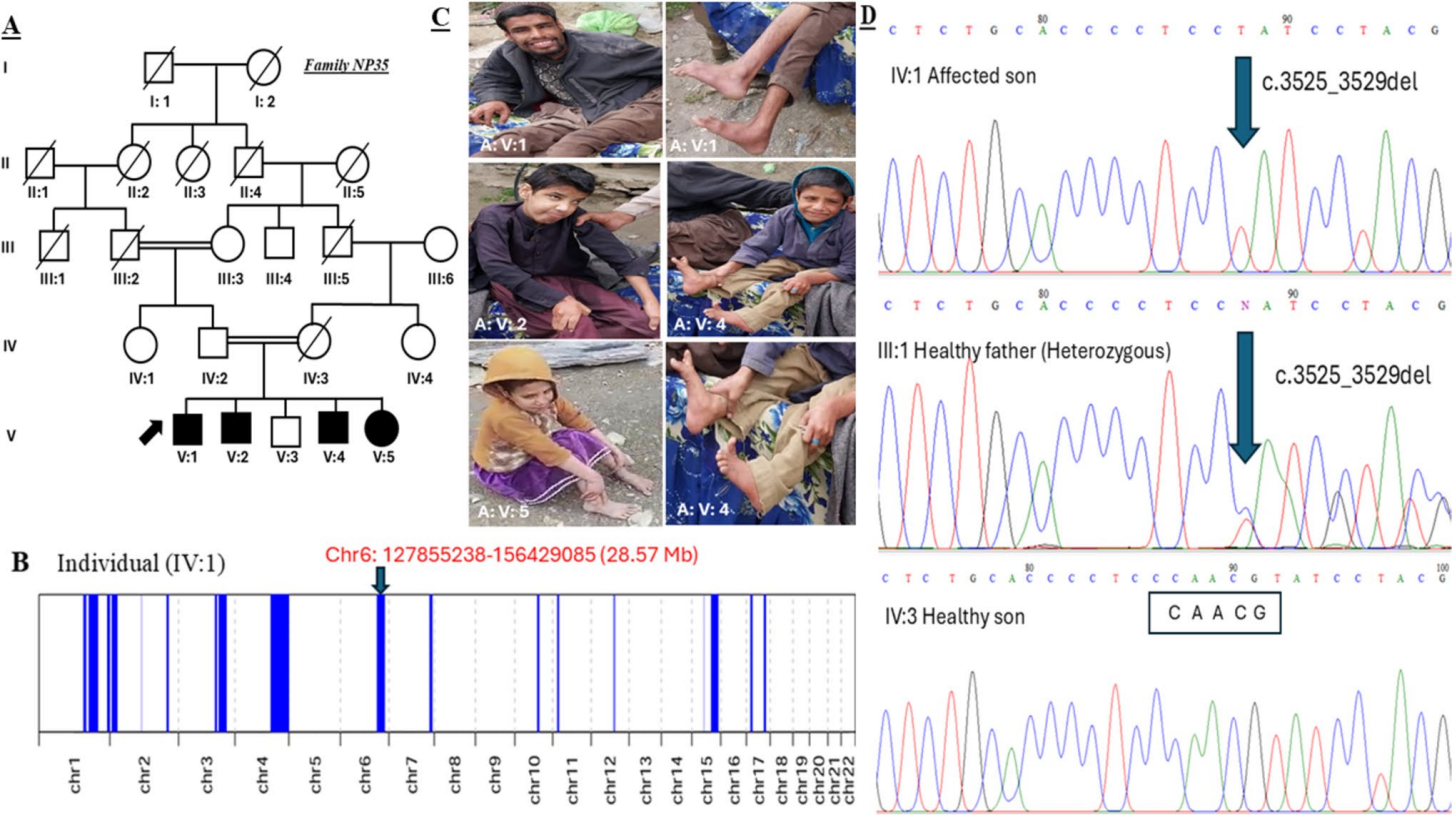
(**A**) Pedigree of family NP35. (**B**) Runs of homozygosity regions. (**C**) Clinical presentation of the patient. (**D**) Electropherograms representing segregation of identified variant in the family

**Fig. 2:**
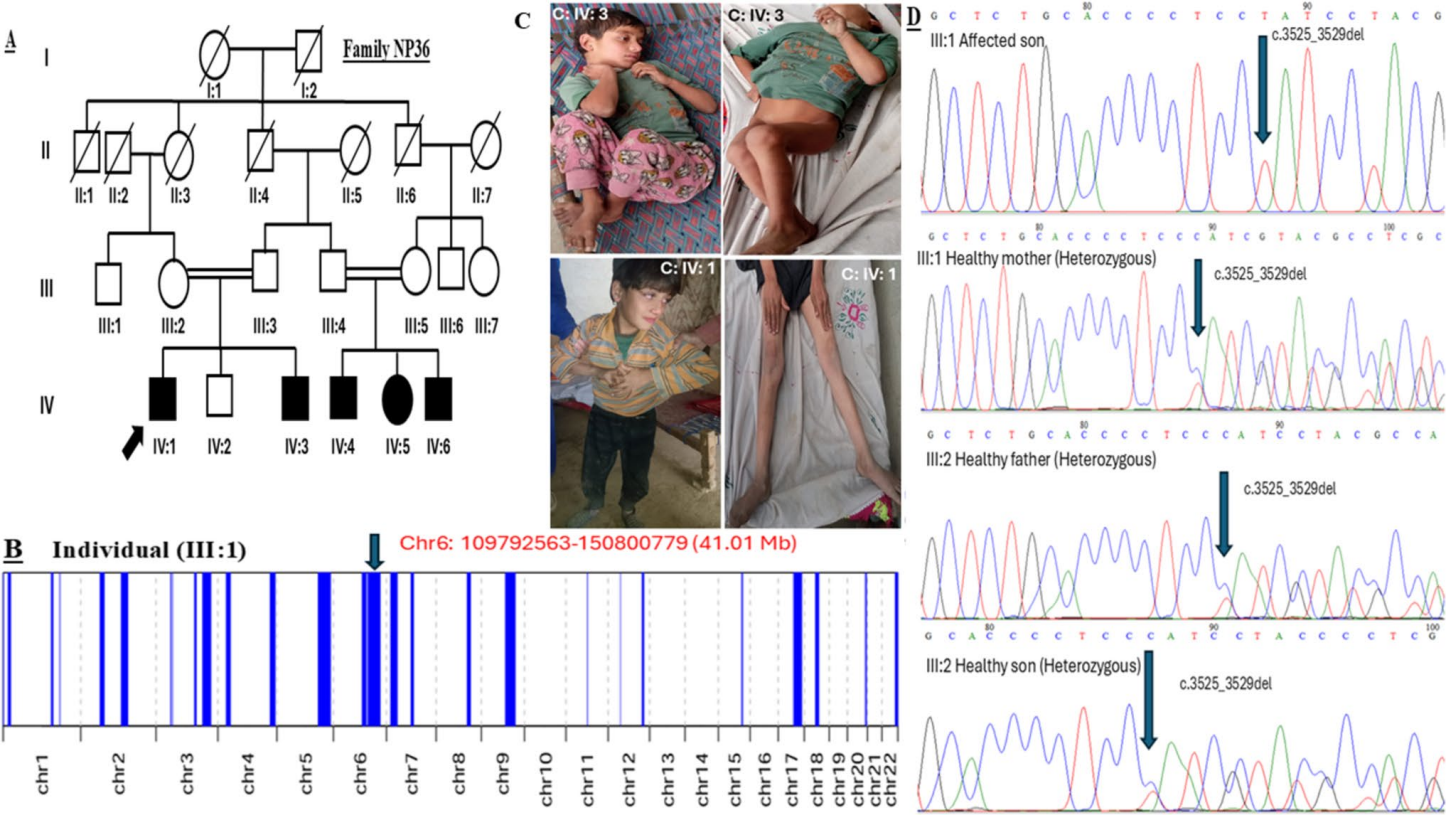
(**A**) Pedigree of family NP36. (**B**) Runs of homozygosity regions. (**C**) Clinical presentation of the patient. (**D**) Electropherograms representing segregation of identified variant in the family

Primer3Web (version 4.1.0) tool (https://primer3.ut.ee/) was used as described previously [5] for primer designing and the target sequence relevant to the candidate gene variant was amplified through PCR. A pair of forward primer (AACTGACCCCGGATGATTCG) and reverse primer (GTGGGAGATCTCTGGCTTGT) was used for PCR amplification under the following conditions: initial denaturation at 96 °C for 8 min, followed by denaturation at 94 °C for 35 s, annealing at 62 °C for 30 s and extension at 72 °C for 45 s. Final PCR products were purified through a kit-based method (GenJet PCR Purification Kit, Thermo Scientific USA). The specific products were assessed via a 2% agarose gel, alongside a 100-base pair ladder (Thermo Scientific USA). Using a standard protocol, the candidate gene (*GRM1*) variant was tested for co-segregation with the disease phenotype using Sanger sequencing (ABI3730 DNA Analyzer). The results provided by the ABI3730 DNA Analyzer were visualized and analyzed with BioEdit Sequence Alignment Editor version 7.2.6. For homozygosity mapping, an online tool named AutoMap (https://automap.iob.ch/process) was used with default settings, which needed a VCF file generated by whole exome sequencing to show the homozygous regions (Fig. 1B and Fig. 2B).

## Results

We identified a novel *GRM1* variant (NM_001278064.2):c.3525_3529del in both families. This variant predicts a frameshift causing the substitution of asparagine at position p.1176 with isoleucine, followed by a premature stop codon 71 amino acids downstream [p.(Asn1176IlefsTer71)] and a truncated protein.

### Clinical Reports

Table 1 summarizes the most consistent clinical findings of all affected individuals of the two families (NP35 and NP36). The pedigrees of the families and clinical photographs of the affected individuals are presented in Fig. 1 and Fig. 2. In both families, the identified variant was found segregated in an autosomal recessive pattern (Fig. 1D and Fig. 2D).

A five-generation consanguineous family (NP35) was comprised of four affected individuals (V:1, V:2, V:4 and V:5) and one healthy individual (V:3) who were born after a normal pregnancy (Fig. 1A). Parents of the affected family were healthy/asymptomatic. Clinical symptoms were observed in infancy in all affected subjects, like feeding difficulties and developmental delay. Karyotyping analysis was normal for the subject (V:1) at the age of one year. Multiple biochemical tests gave normal results as confirmed by recent clinical evaluation. In the affected individuals, the common clinical features were psychomotor deficits, lack of ambulation, motor dysfunction, severe intellectual disability, jerky and roving eye movements, inability to eat, severe speech delay, frequent falls and dystonic tremors. Interestingly, subjects other than V:1 exhibited severe repetitive jerks or seizures. The clinical manifestations closely matched with SCAR13 phenotype (OMIM: 614,831).

A four-generation consanguineous family (NP36) was affected with SCAR13 in an autosomal recessive pattern of inheritance, with five affected individuals who presented disease onset at the age of 1–2 years of life (Fig. 2A). Clinical history in all affected individuals of the family was consistent. The proband (IV:1) presented with severe intellectual disability, dysmetria, involuntary movements, speech delay, gait ataxia and frequent falls. No hearing or cognitive impairment was observed in this family. The affected individual IV:3 had a normal chromosomal karyotype, and several other laboratory parameters, like complete blood counts, liver function test, and serum electrolytes, were also normal. Based on clinical and physical examinations, it was suggested a case of SCAR13.

The phenotypes of both families overlapped, but the family NP35 was uniquely severe in terms of clinical manifestations such as seizures, developmental delay and jerky and roving eye movements.

## Discussion

We investigated nine SCAR13 patients from two families, and identified a novel pathogenic *GRM1* frameshift variant c.3525_3529; p.(Asn1176IlefsTer71) in C-terminal domain (CTD) of mGluR1 (alpha isoform), segregating in an autosomal recessive pattern of inheritance. Variants in CTD of mGluR1 are not previously reported in SCAR13 cases, however, a de novo heterozygous frameshift variant p.Gly1056Argfs*49 in CTD was found in the proband of a family affected with autosomal dominant SCA44. This variant exhibited a dominant-negative effect associated with juvenile-onset ataxia and intellectual disability [29].

The variant c.3525_3529; p.(Asn1176IlefsTer71) is present in the last exon of *GRM1* that may be predicted to escape nonsense mediated decay and would result in the production of a truncated mGluR1 in consistence with previous report [29] that of C-terminal *GRM1* variant. The truncation may exert dominant negative effects via dimerizing with wild type mGluR1, disrupting synaptic transmission. Alternatively, it could mislocalize to the cytoplasm, disrupting cerebellar circuit formation. In murine models having CTD deletions exhibit ataxia and synaptic defects [22], mirroring SCAR13 phenotypes.

Our identified variant is classified as a variant of uncertain significance (VUS) according to the ACMG guidelines. Uniquely, the same variant led to more severe clinical manifestations in the patients of family NP35 than those of family NP36, as mentioned in Table 2 (video is available upon request). So, this study presents a novel variant with interfamilial clinical heterogeneity suggesting the involvement of additional molecular modifiers and a more complex genotype–phenotype correlation.

**Table 2:** Comparison of clinical presentation of two families affected with SCAR13 included in this study

Feature	Family NP35	Family NP36
Patient IDs	V:1, V:2, V:4, V:5	IV:1, IV:3, IV:4, IV:5, IV:6
Gender	3 Male, 1 Female	4 Male, 1 Female
Current Age	6 to 22 years	5 to 10 years
Onset Age	1 year	1–2 years
OMIM Syndrome	OMIM: 614831	OMIM: 614831
Developmental delay	Yes, Severe	Mild to moderate
Motor Function	Inability to stand or walk	Inability to stand, gait ataxia
Oculomotor Symptoms	Severe nystagmus, jerky and roving eye movements	No
Eye ptosis	Yes	No
Speech impairment	Yes, Severe	Yes, Moderate
Intellectual Disability	Yes, Severe	Mild to moderate
Feeding difficulty	Yes	Yes
Other Neurological Signs	Dystonic tremors, epilepsy, frequent falls (V:2, V:4, V:5)	Mild involuntary movement, frequent falls
Pyramidal Signs	Yes	Yes
Microcephaly	Mildly observed	No
Scoliosis	Yes	No
Facial dysmorphism	Mild to moderate	No
Brain MRI	NA	NA

Clinical features of the affected individuals within each family were not completely identical but most of their features were consistent

**Fig. 3:**
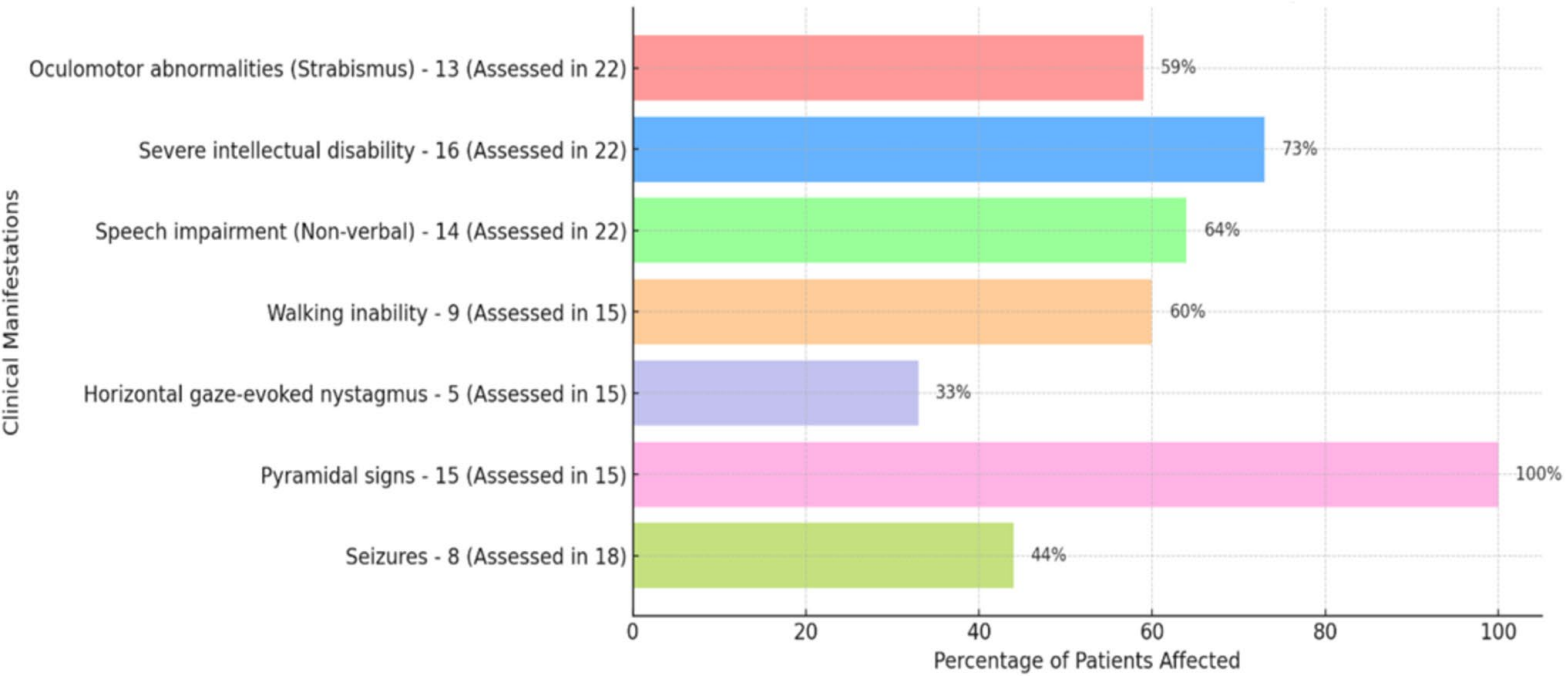
Bar chart illustrating clinical features in the previously reported cases of SCAR13 (n-22)

We reviewed 22 reported patients affected with *GRM1*-associated SCAR13 disorder as summarized in Table 1 [7, 8, 10, 16, 23, 24, 31]. Among these documented SCAR13 patients (n = 22), 13 (59%) were observed with oculomotor abnormalities (strabismus). Severe intellectual disability was observed in 16 (73%) patients while speech impairment was found in 14 (64%) patients who were nonverbal. Clinical manifestations of walking inability, horizontal gaze-evoked nystagmus, and pyramidal signs each were assessed in 15 cases and recognized in 9 (60%), 5 (33%) and 15 (100%) patients, respectively. Presence of seizures was evaluated in 18 cases and found in 8 (44%) patients (Fig. 3). Most clinical symptoms of our investigated families overlap with those described by Guergueltcheva et al. [16], Davarniya et al. [10] and Yousaf et al. [31] (Table 1). Scoliosis has been previously reported in a single patient only [31]. In our family NP35, all four affected individuals exhibited scoliosis, while it was absent in the patients of family NP36.

Disease severity in SCAR13 is considered a function of the type of variant in the patients. In the two published reports, the frameshift variant led to truncation of the receptor protein that was considered responsible for severe phenotypes in the affected individuals [7, 16]. However, both of our families were identified with the same frameshift variant presenting interfamilial clinical heterogeneity,family NP35 had severe phenotype than the family NP36. Our study indicates that the variation in the clinical manifestations and severity involves the presence of modifier genes in addition to the type of mutations. Investigations are recommended to further elucidate the molecular basis of SCAR13 including the potential modifier genes.

Other than spinocerebellar ataxia, there are published data on the *GRM1* variants associated with increased risk of autism, schizophrenia, bipolar disorder, and attention deficit hyperactivity disorder [4, 9, 13, 15, 26, 27]. This pleiotropy underscores the mGluR1 role in synaptic plasticity and neurodevelopment with tissue-specific isoforms. However, in our families, symptoms associated with these disorders were not observed.

Our investigation led to expand the number of SCAR13 cases from 22 to 31 in the literature and reported a novel *GRM1* variant with clinical heterogeneity. Furthermore, this is the first report of SCAR13 case with a variant in mGluR1 C-terminal domain. This study reinforces the evidence of disrupted mGluR1 signalling in cerebellar dysfunction leading to early onset of SCAR13. Moreover, features like scoliosis and epilepsy are rarely observed in SCAR13 and should be considered in future clinical and research studies.

## Supplementary Information

Below is the link to the electronic supplementary material.

ESM 1 Filtration criteria applied in the current study for variant detection in the exome sequencing (PNG 123 KB)

High Resolution Image (TIF 1.62 MB)

## Data Availability

No datasets were generated or analysed during the current study.
