# Supplementary figures and images for: Identification of a Novel GRM1 Frameshift Variant in Two Pakistani Families Broadens the Genetic Landscape of Ultra-Rare Spinocerebellar Ataxia Type 13
Source: Cerebellum. 2025 Aug 27;24(5):145. doi: 10.1007/s12311-025-01897-w (PMC12380963; doi:10.1007/s12311-025-01897-w)

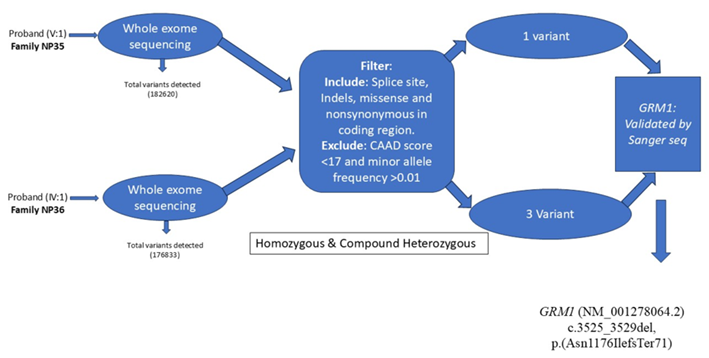

Supplement: Supplementary file 1 — Filtration criteria applied in the current study for variant detection in the exome sequencing (PNG 123 KB) [file 12311_2025_1897_Fig4_ESM.png]

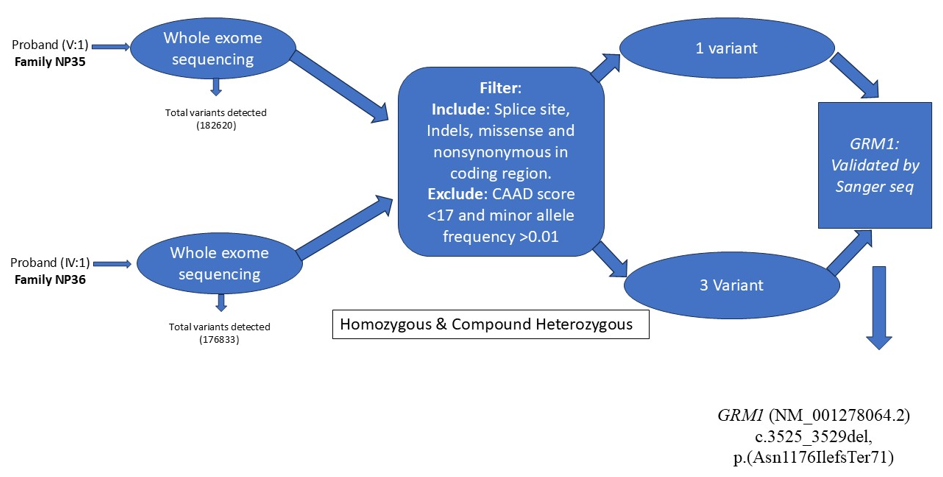

Supplement: Supplementary file 2 — High Resolution Image (TIF 1.62 MB) [file 12311_2025_1897_MOESM1_ESM.tif]
